# Subacute Meningitis: A Rare Presentation of Brucellosis

**DOI:** 10.7759/cureus.70445

**Published:** 2024-09-29

**Authors:** Mafalda Duarte, Vasco Tiago, Raquel Sousa, Fernando Aldomiro

**Affiliations:** 1 Internal Medicine, Hospital Prof. Doutor Fernando Fonseca, Amadora, PRT; 2 Internal Medicine, Hospital Dr. José de Almeida, Cascais, PRT; 3 Internal Medicine, Hospital Lusíadas Amadora, Amadora, PRT

**Keywords:** brucellosis, fever, meningitis, weight loss, zoonotic infections

## Abstract

A young male adult presented with a two-week-long history of myoarthralgia, vomiting, febrile sensation, holocranial headache with photophobia, neck stiffness, anorexia, and weight loss. He worked as a butcher in a local slaughterhouse, and two of his coworkers had recently been diagnosed with brucellosis. On his fourth visit to the Emergency Department (ED), fever was observed for the first time. He was admitted for subacute meningitis. A lumbar puncture revealed high protein concentration, low glucose concentration, and pleocytosis without predominance. He was started on empirical treatment for acute meningitis and neurobrucellosis with ceftriaxone, doxycycline, and rifampin. There was a complete symptom remission over one week. The etiological investigation yielded a positive cerebrospinal fluid (CSF) and serum Rose Bengal Tests (RBTs), as well as a positive anti-*Brucella* IgG titer, leading to a final diagnosis of neurobrucellosis. After nine months of antimicrobial therapy and two years of follow-up, the patient remained asymptomatic.

## Introduction

Subacute meningitis is most often caused by *Mycobacterium tuberculosis* or *Cryptococcus neoformans*. More rarely, other infectious agents or even noninfectious pathologies may present as subacute or chronic meningitis [1]. Therefore, a correct diagnosis can sometimes be challenging.

Brucellosis is an infectious disease caused by gram-negative bacilli *Brucella *species, which are easily aerosolized and have a low infectious dose and a prolonged incubation period. In Europe, brucellosis is relatively common in the southernmost countries, namely, Spain and Portugal, where it is one of the most common zoonoses [2]. In endemic countries, brucellosis is an important cause of subacute and chronic meningitis, although this is a relatively rare presentation of the disease [3].

In the past few years, several neurobrucellosis case series have been published, especially in areas with high incidence rates such as the Mediterranean Basin, Eastern Europe, Asia, Central and South America, Africa, and the Middle East [4-7]. In these analyses, the clinical picture has been variable, although some symptoms (headache, fever, myalgias, sweating, low back pain, weight loss) and signs (changes in behavior, meningeal signs) are relatively constant. Most of these signs are common in systemic brucellosis without neurological involvement, but in neurobrucellosis, there is usually focal neurological signs excluding other etiologies. Headache, hearing loss, confusion, behavioral changes, and meningeal signs are the main signs and symptoms suggesting neurobrucellosis rather than systemic brucellosis.

Most of brucellosis cases are foodborne and occur after consumption of unpasteurized dairy products or raw or undercooked pork [3,8-10]. As such, brucellosis is an important diagnosis to consider in the setting of subacute meningitis in an endemic area, especially when there is a history of exposure or risk factors.

## Case presentation

We present a case of a 23-year-old man with a known history of migraines who worked as a butcher in a local slaughterhouse. He appealed to a family doctor consultation due to a two-week history of shoulder girdle myalgia, arthralgia, vomiting with epigastric pain, unquantified fever sensation, neck stiffness, and holocranial headache with photophobia. He also reported anorexia and unquantified weight loss. Two weeks before this incident, the patient experienced a few episodes of night sweats without any other associated symptoms. Two of his work colleagues had been diagnosed with brucellosis in the past three years, with one also reporting neck stiffness and holocranial headache with photophobia, arthralgia, and fever sensation.

**Figure 1:**
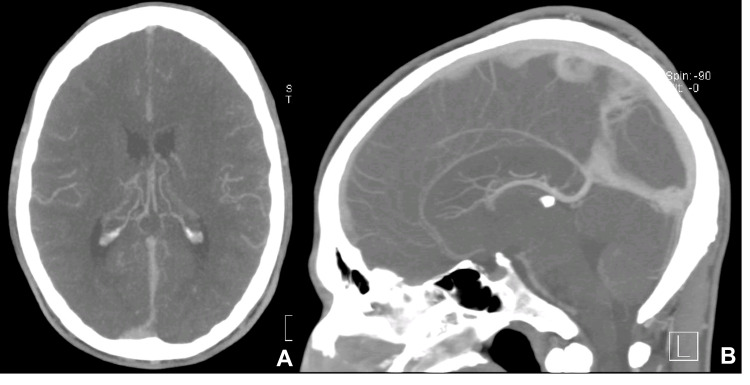
CT cerebral venous angiography without changes CT: Computed tomography (A) Axial CT cerebral venous angiography section. (B) Sagittal CT cerebral venous angiography section

He was taken to the local hospital's Emergency Department (ED) for evaluation. The objective examination was normal. This examination included a summary neurological examination (without meningeal or focal neurological signs) and an assessment of the osteoarticular apparatus. Laboratory studies, including complete blood count, renal function, electrolytes, liver tests, C-reactive protein, amylase, glucose, and creatine kinase, showed no abnormalities. He was discharged with symptomatic treatment for migraine. In the following days, he returned to the ED twice with worsening symptoms. A cranioencephalic computed tomography (CT) and a CT cerebral venous angiography (Figure 1) were performed without changes, excluding tumors, structural anomalies, and venous thrombosis.

At this stage, the family doctor contacted the hospital team directly to inform them of the clinical suspicion of neurobrucellosis. The patient was referred to the ED once again and presented with confusion and fever (38.7ºC) at hospital admission. A lumbar puncture (LP) was performed with the results presented in Table 1 showing elevated protein levels, low glucose levels, and pleocytosis. The remainder of laboratory profile did not show abnormalities.

**Table 1:** Analysis of cerebrospinal fluid on admission In the second evaluation (in the third week of treatment), the cell count showed a predominance of mononuclear cells (99%)

Cerebrospinal fluid characteristics	Results
Aspect	Clear
Protein	359 mg/dL
Glucose	19 mg/dL
Cell count	276/μL (no predominance)
Direct examination	Negative
Cultural examination	Negative
Capsular antigens	Negative

Based on epidemiological data, empirical therapy with ceftriaxone (for acute bacterial meningitis and neurobrucellosis) and doxycycline and rifampicin (for neurobrucellosis) was started. There was a complete symptomatic resolution within a week.

Table 2 shows a summary of the etiological investigation. The Rose Bengal Test (RBT) in cerebrospinal fluid (CSF) and blood were positive (unquantified titer). Serum serology for *Brucella* was positive for anti-*Brucella* IgG (enzyme-linked immunosorbent assay) but negative for IgM. Both polymerase chain reaction (PCR) and culture of blood and CSF were negative for *Brucella*. All other targeted tests were negative, except for weakly positive serologies for *Rickettsia conorii* and *Coxiella burnetii* phase I.

**Table 2:** Etiological findings EBNA: Epstein-Barr nuclear virus antigen 1; EIA: enzyme-linked immunosorbent assay; IGRA: interferon gamma release assay; CSF: cerebrospinal fluid; PCR: polymerase chain reaction; AU: arbitrary units; VCA: viral capsid antigen; VDRL: Venereal Disease Research Laboratory; HIV: human immunodeficiency virus Reference ranges: Cytomegalovirus IgG < 12U/mL; *Rickettsia conorii* IgG < 40 AU/mL; *Brucella* spp IgG (EIE) < 20 AU/mL; *Coxiella burnetii* IgG phase I < 11 AU/mL

Laboratory studies	Results
Anti-HIV1/anti-HIV2/p24	Negative
Herpes simplex multiplex PCR	Negative
Toxoplasma	
IgM	Negative
IgG	Negative
Mycoplasma	
IgM	Negative
IgG	Negative
Epstein-Barr virus	
Anti-VCA IgM	Negative
Anti-VCA IgG	Negative
Anti-EBNA	Negative
Cytomegalovirus	
IgM	Negative
IgG	132 U/mL
VDRL	Negative
IGRA	Negative
Rickettsia conorii	
IgM	Negative
IgG	80 AU/mL
*Borrelia *spp.	
IgM	Negative
IgG	Negative
*Brucella* spp.	
Rose Bengal Test (blood, CSF)	Positive (unquantified titer)
IgM (EIA)	Negative
IgG (EIA)	> 200 AU/mL
Culture (blood, CSF)	Negative
PCR (serum, CSF)	Negative
Coxiella burnetii	
IgM phase I	Negative
IgG phase I	19 AU/mL
IgM phase II	Negative
IgG phase II	Negative

The National Epidemiological Surveillance System (SINAVE) criteria [8] (arthralgia, night sweats, headache, anorexia, and positive serum serology for *Brucella*) and the WHO criteria [9] (history of exposure to a known source of *Brucella, *night sweats, neck stiffness, headache, and positive serum serology for *Brucella*) for a confirmed diagnosis were met. However, because the IgG titers were immeasurably high (>200 AU/mL) from the beginning, it was impossible to observe a quadrupling of the titer, so the Centers for Disease Control and Prevention (CDC) criteria for a definitive diagnosis were not met [10]. The CDC criteria for a presumptive diagnosis were also not met because a standardized agglutination test was not performed [10].

**Figure 2:**
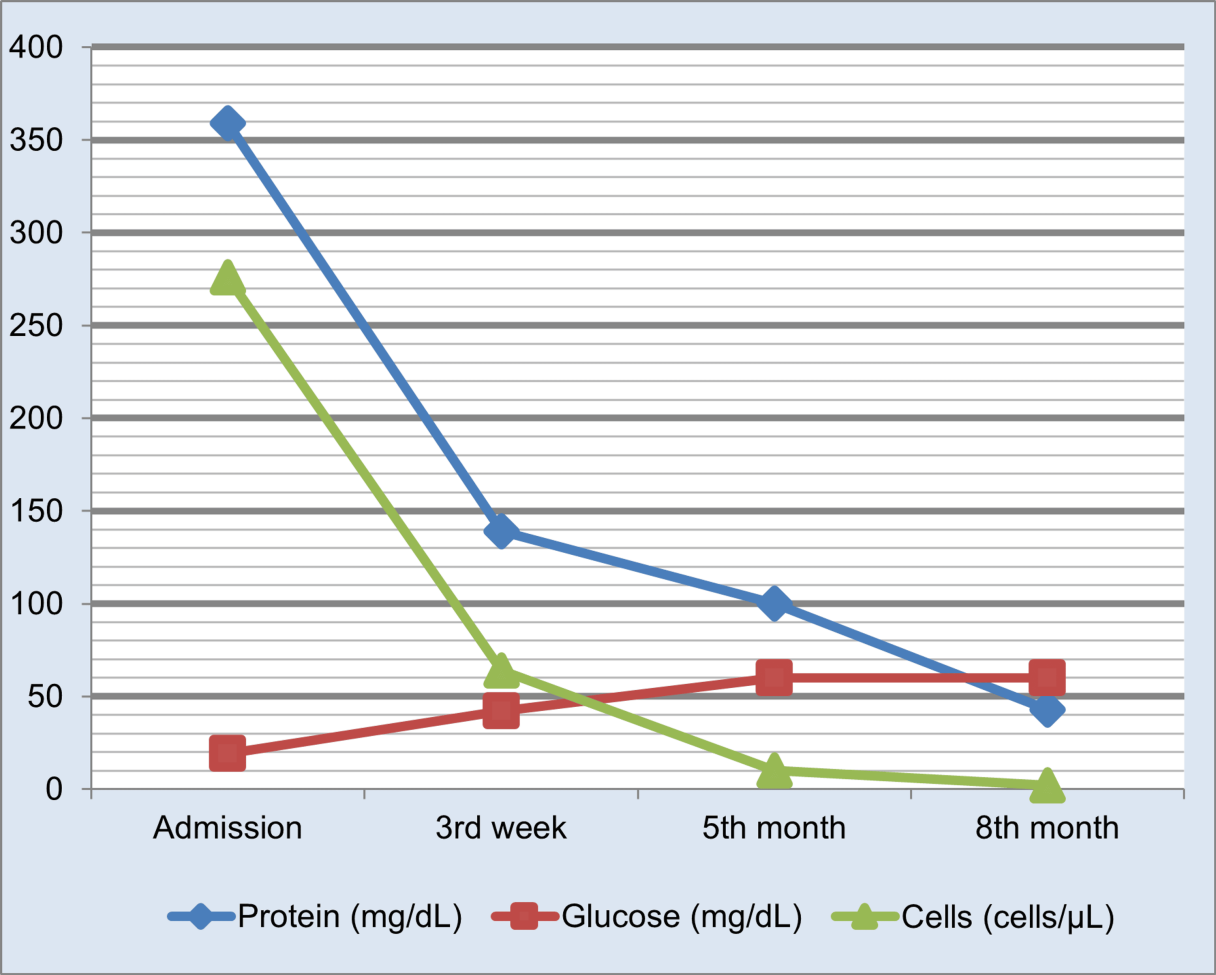
Evolution of the cytochemical examination of cerebrospinal fluid Reference ranges: protein 15-45 mg/ dL; glucose 40-70mg/ dL; cell count less than 5/µL

After confirming the diagnosis of neurobrucellosis, antibiotic therapy with ceftriaxone was continued for three weeks, in addition to doxycycline and rifampicin, which was continued thereafter. At this point, a repeated LP demonstrated improvement but not complete remission (Figure 2) with a predominance of mononuclear cells (99%). The patient was discharged from the hospital, and the remaining treatment was carried out with oral doxycycline and rifampicin. A total of nine months of antibiotic therapy was completed, according to the evolution of the findings of the CSF cytochemical examination which normalized in the eighth month (Figure 2). No other antibiotic therapy was administered, and no further supportive treatments were necessary. After two years of follow-up, there was no evidence of relapse, so the patient is considered cured.

## Discussion

Brucellosis is an increasingly rare disease in Portugal with an incidence of 0.09 cases per 100 000 population in 2020 [11,12]. This is due to the prevention measures put in place such as mandatory pasteurization for all milk and milk products and specific protection measures to reduce occupational risk. Infection of the central nervous system is even rarer, occurring in only approximately 10% of cases [3]. Our case differs from the others due to the reported location of the myalgias (shoulder girdle), but all other symptoms are consistent with the most common manifestations of neurobrucellosis.

The combination of exposure history, clinical picture, and positive anti-*Brucella* titer IgG is highly suggestive of brucellosis. Serological tests are the most commonly used method for diagnosing brucellosis, as they provide the most significant number of positive results while maintaining high specificity [13]. A high serum IgG antibody titer and positive CSF RBT are considered very reliable for neurobrucellosis’ diagnosis [13-15]. Excessive IgG antibody has been described as a cause for a negative IgM titer in patients with brucellosis [16]. Alternatively, this condition could be a reactivation of chronic brucellosis, which could also present with a negative IgM titer [10].

On the other hand, molecular methods and cultures are also important as they provide more reliable confirmation, although their diagnostic accuracy is lower. Culture, which is the reference method for diagnosis, has relatively low sensitivity [13], especially in neurobrucellosis [4,7,13]. Positive PCR is a criterion for a presumptive diagnosis according to CDC standards [6], but its sensitivity varies according to the quality of the sample [13].

As described above, this case met the criteria for definitive diagnosis proposed by SINAVE [8] and the WHO [9], but not those proposed by the CDC [10], which were impossible to meet from the start due to the initial high antibody titer. Additionally, the criteria for presumptive diagnosis were also not met, possibly due to its inherent limitations in reactivated chronic brucellosis, which could be the case. A standardized agglutination test was not performed, but in chronic brucellosis, its diagnostic accuracy is low, and immunoenzymatic assays have better performance. Likewise, cultures and PCR in chronic brucellosis also have low sensitivity [10,13,16]. The weakly positive serologies for *Rickettsia conorii* and *Coxiella burnetii *were considered to be either cross-reactions or indicative of past exposure. This interpretation was based on the clinical presentation and etiological findings, which were more consistent with brucellosis. Additionally, only IgG titers were positive.

## Conclusions

We have presented a case of neurobrucellosis where there was an occupational history crucially pointing to this diagnosis, which is a rare presentation of an uncommon infection. Active listening to the patient and their family as well as direct contact with the hospital team, were also key elements.

The targeted treatment led to a gradual and progressive improvement in symptoms, lagging behind improvement of CSF cytochemical examination, further supporting this diagnosis. Notably, the only positive laboratory finding was serology, which is common in chronic brucellosis and neurobrucellosis cases where cultures and PCR tests have low sensitivity.

## References

[1] Zunt JR, Baldwin KJ (2012). Chronic and subacute meningitis. Continuum (Minneap Minn).

[2] Pappas G, Papadimitriou P, Akritidis N, Christou L, Tsianos EV (2006). The new global map of human brucellosis. Lancet.

[3] Gul H, Erdem H (2014). Brucellosis (Brucella species). Mandell, Douglas, and Bennett's Principles and Practice of Infectious Diseases.

[4] Gul HC, Erdem H, Bek S (2009). Overview of neurobrucellosis: a pooled analysis of 187 cases. Int J Infect Dis.

[5] Dreshaj S, Shala N, Dreshaj G, Ramadani N, Ponosheci A (2016). Clinical manifestations in 82 neurobrucellosis patients from Kosovo. Mater Sociomed.

[6] Tajerian A, Sofian M, Zarinfar N, Ramezani A (2024). Manifestations, complications, and treatment of neurobrucellosis: a systematic review and meta-analysis. Int J Neurosci.

[7] Naderi H, Sheybani F, Parsa A, Haddad M, Khoroushi F (2022). Neurobrucellosis: report of 54 cases. Trop Med Health.

[8] (2015). National Epidemiological Surveillance System (SINAVE)-guidance for health authorities and public health units. https://sinave.min-saude.pt/SINAVE.MIN-SAUDE/despacho_n_15385-A-2016.

[9] Corbel M, Alton G, Ariza J (2006). Brucellosis in humans and animals. https://www.moh.gov.bt/wp-content/uploads/afd-files/2014/11/Brucellosis-WHO-gudeline.pdf..

[10] (2017). Brucellosis reference guide: exposure, testing, and prevention. https://www.cdc.gov/brucellosis/pdf/brucellosi-reference-guide.pdf.

[11] Gaspar CG, Augusto GF, Albuquerque MJ, Nascimento MR, Vicêncio PO, Nogueira P (2017). Mandatory reporting diseases 2013-2016. Volume I-Portugal. Mandatory reporting diseases 2013-2016. Volume I - Portugal.

[12] (2022). Annual epidemiological report for 2020: brucellosis. https://www.ecdc.europa.eu/sites/default/files/documents/AER_2020_Report_BRUC.pdf.

[13] Araj GF (2010). Update on laboratory diagnosis of human brucellosis. Int J Antimicrob Agents.

[14] Guven T, Ugurlu K, Ergonul O (2013). Neurobrucellosis: clinical and diagnostic features. Clin Infect Dis.

[15] Díaz R, Casanova A, Ariza J, Moriyón I (2011). The Rose Bengal Test in human brucellosis: a neglected test for the diagnosis of a neglected disease. PLoS Negl Trop Dis.

[16] Sharma R, Chisnall C, Cooke RP (2008). Evaluation of in-house and commercial immunoassays for the sero-diagnosis of brucellosis in a non-endemic low prevalence population. J Infect.

